# Racemization in Reverse: Evidence that D-Amino Acid Toxicity on Earth Is Controlled by Bacteria with Racemases

**DOI:** 10.1371/journal.pone.0092101

**Published:** 2014-03-19

**Authors:** Gaosen Zhang, Henry J. Sun

**Affiliations:** Division of Earth and Ecosystem Sciences, Desert Research Institute, Las Vegas, Nevada, United States of America; RMIT University, Australia

## Abstract

D-amino acids are toxic for life on Earth. Yet, they form constantly due to geochemical racemization and bacterial growth (the cell walls of which contain D-amino acids), raising the fundamental question of how they ultimately are recycled. This study provides evidence that bacteria use D-amino acids as a source of nitrogen by running enzymatic racemization in reverse. Consequently, when soils are inundated with racemic amino acids, resident bacteria consume D- as well as L-enantiomers, either simultaneously or sequentially depending on the level of their racemase activity. Bacteria thus protect life on Earth by keeping environments D-amino acid free.

## Introduction

With the exception of glycine, all amino acids are chiral and may exist as levorotatory (L) or dextrorotatory (D) enantiomer. At Earth's surface temperatures amino acids spontaneously racemize, converting from one form to another [1]. Consequently, although on Earth organisms only synthesize L-enantiomers, the appearance of D-enantiomers is inevitable, especially in soils and sediments where amino acids are sequestered [1], [2]. Additionally, in some organisms, racemization is enzymatically catalyzed (i.e. by racemases), and the resultant D-amino acids are incorporated into non-protein molecules. Such molecules include peptide antibiotics [3], siderophores [4], surfactins [5], and peptidoglycans [6]. The latter, which contain D-alanine, D-glutamic acid, and occasionally D-aspartic acid as well, are ubiquitous in bacterial and cyanobacterial cell walls. Relative to proteins, heterochiral peptides are recalcitrant [7], [8]. As a result, oceans, sediments, and soils are enriched in peptide-bound D-amino acids [9]–[17]. In North American grassland soils, for instance, more than 10% of alanine, glutamic acid, aspartic acid, and leucine exist in D-forms [18].

The fate of D-amino acids during matter cycling is unknown. Early work considered the possibility of their utilization as a source of nitrogen by eukaryotes. Many eukaryotes possess D-amino acid oxidases (DAAOs), a detoxification enzyme that controls endogenous D-amino acids [19], [20]. In the presence of DAAOs and molecular oxygen, D-enantiomers, but not L-enantiomers, are destroyed by oxidative deamination. Of the three products that result, hydrogen peroxide is detoxified by catalases, while α-keto acid and ammonium may be metabolized as carbon and nitrogen sources. When exposed to external D-amino acids, however, only yeasts and fungi up-regulate DAAOs fast enough to turn toxicity into nutrient [21]–[25]. Plants and mammals appear to lack such capacity and, as a result, suffer stunted growth [26]–[28].

Studies of *Methanococcus maripaludis*, an archaeon, and *Schizosaccharomyces pombe*, a yeast showed that enzymatic racemization can be coupled to L-amino acid catabolism as a more efficient way of catabolizing D-amino acids [29], [30]. In both organisms, this capability is because of an inducible operon. The operon consists of three genes acquired from bacteria via lateral transfer that specify alanine permease, alanine racemase, and L-alanine dehydrogenase. The permease imports L- and D-alanine. Therefore, for *M*. *maripaludis* and *S*. *pombe*, D-alanine is essentially a source of L-alanine.

Here we provide evidence that all bacteria are capable of reverse racemization. Specifically, we show that when soils are inundated with LD-alanine, LD-aspartic acid, LD-glutamic acid, and LD-leucine, resident bacteria absorb L- and D-enantiomers in equal or nearly equal rates. In the case of alanine, the conversion of D-enantiomers appears to be enabled by constitutive alanine racemases that are ordinarily anabolic in function (i.e. cell wall synthesis). In the case of the other three amino acids, the exposure to L-enantiomers appears to induce catabolic racemases. As a result, as soon as L-enantiomers are exhausted, the organisms could consume D-enantiomers.

## Results

A simple nutrient solution containing 50 mM of D-glucose as a carbon source and 4 mM of LD-alanine, LD-aspartic acid, LD-glutamic acid, and LD-leucine as a nitrogen source was inoculated with soils. The soils originated from South and North American semiarid deserts, alpine forests, wetlands, and landscape lawns. They contained between 1×10^6^ and 6×10^6^ colony-forming units (CFU) of bacteria per gram soil. The ratio of soil and medium was 1∶2 (w∶v). The soil suspensions were incubated at 20°C under aerobic conditions.

**Figure 1:**
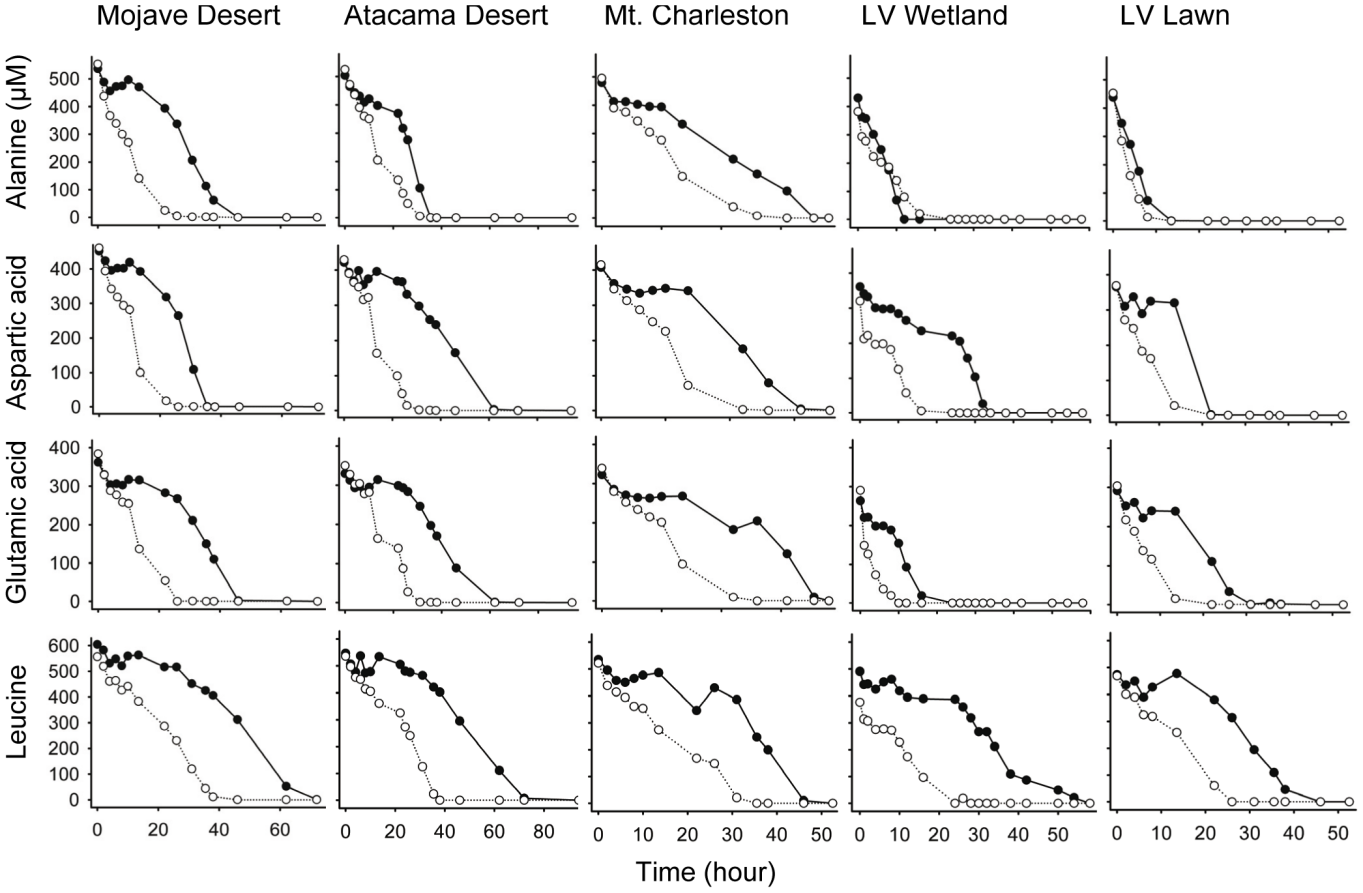
Microbial consumption dynamics of racemic amino acids (L-enantiomers: open symbol; D-enantiomers: filled symbol) following addition to soils. The rapid absorption of D-enantiomers is presumably by bacteria, which possess amino acid racemases and can turn D-enantiomers into L-forms.

The ensuing absorption of amino acid enantiomers followed two patterns (Figure 1). For wetland and lawn soils, D- and L-alanine were consumed simultaneously and in equal rates. In all other cases, L- and D-enantiomers were consumed one after another. More specifically, the consumption of D-enantiomers occurred in three phases: a transient initial decline, an intervening phase of stasis, and a final phase of rapid utilization. Only the first phase was observed in sterile (autoclaved) soils, indicating that it is abiotic in nature (Figure S1). During the course of the experiment, the numbers of bacteria in the samples doubled 5–7 times.

**Figure 2:**
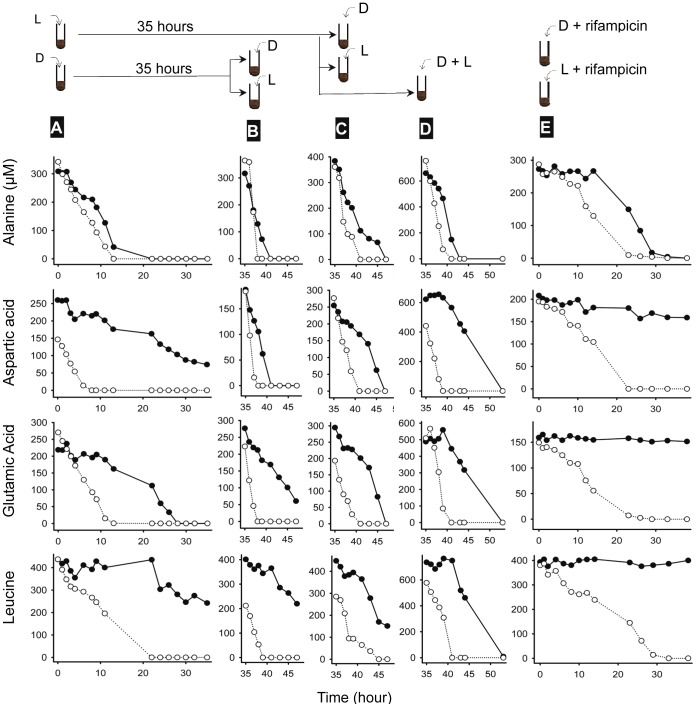
Microbial consumption dynamics of amino acids (L-enantiomers: open symbol; D-enantiomers: filled symbol) by a Mojave Desert soil. Amino acids were added under four different conditions: A) first injections containing L- or D-enantiomers; B) and C) second injections containing L- or D- enantiomers; D) second injections containing racemic mixtures; and E) first injections containing L- or D-enantiomers and 25 mM of rifampicin, an antibiotic that inhibits RNA synthesis. For bacteria to catabolize D-enantiomers two conditions must be met: 1) racemases are induced if not already present due to anabolic functions, e.g. alanine racemase, and 2) L-enantiomers are exhausted.

To understand the consumption mechanisms, we studied the Mojave Desert soil and *Arthrobacter* sp., a bacterium isolated from the Atacama Desert, in more detail. When the soil received only D-enantiomers, consumption dynamics changed. In the case of D-alanine, absorption began immediately and was nearly identical in rate to that of L-alanine, determined in a separate sample (Figure 2A). Clearly, the organisms were capable of consuming D-alanine, but did not do so if L-alanine were available.

Without L-enantiomers, absorption dynamics of D-aspartic acid, D-glutamic acid, and D-leucine also were altered, but in a different fashion. Little or no activity occurred in the first few hours. With further incubation, consumption became progressively faster (Figure 2A). By hour 35, it was comparable to the consumption of corresponding L-enantiomers (Figure 2B). To verify that L-enantiomers induced catabolism of D-enantiomers, we incubated soil in L-enantiomers before adding D-enantiomers. Consumption began immediately and was rapid (Figure 2C). When the same induced soil was given a mixture of L- and D-enantiomers, however, consumption of D- enantiomers began only after L-enantiomers were consumed (Figure 2D).

RNA synthesis and gene expression were essential to utilizing D-aspartic acid, D-glutamic acid, and D-leucine. In the presence of 25 mM of rifampicin, an antibiotic that inhibits RNA synthesis, utilization of these three D-enantiomers was prevented. Consumption of L-enantiomers and D-alanine, in contrast, remained intact, indicating that the failure to use D-aspartic acid, D-glutamic acid, and D-leucine was not due to cell death (Figure 2E).

**Figure 3:**
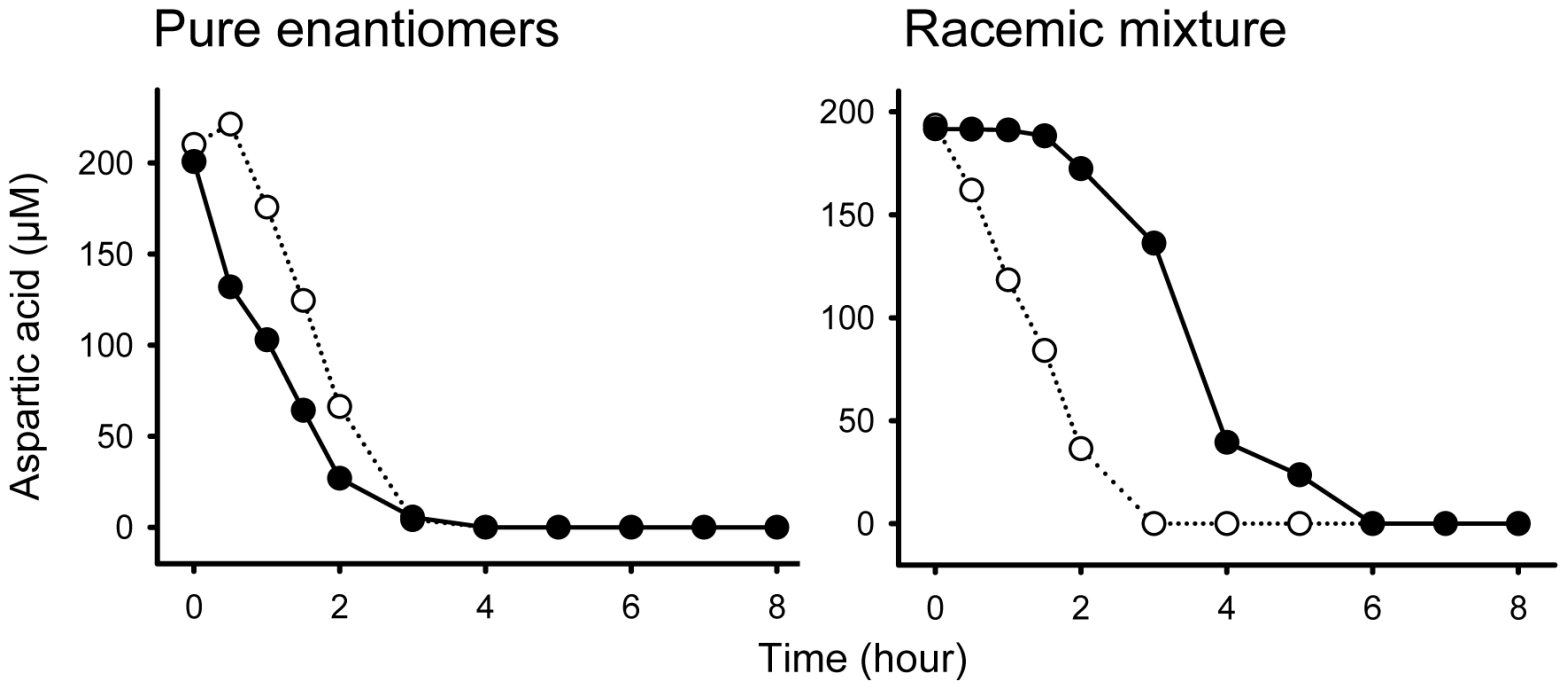
Consumption dynamics of L- (open symbol) and D-aspartic acid (filled symbol) by bacterium *Arthrobacter* sp. when they were added separately and in a racemic mixture.

When D- and L-aspartic acid were added to two *Arthrobacter* sp. cultures (10^8^ CFU/ml) as pure enantiomer preparations, they were consumed identically (Figure 3). When they were added as a mixture, utilization of D-aspartic acid did not begin until after L-aspartic acid was consumed.

## Discussion

Because D-alanine is an essential component of their cell wall [6], [31], [32], all bacteria are expected to synthesize alanine racemases constitutively. Although other D-amino acids are also essential, they do not necessarily require specific racemases to form. For instance, D-glutamic acid may form by racemization or transamination, between D-alanine and L-glutamic acid [33]. Our study suggests that when D-alanine is available externally, alanine racemases are temporarily freed from anabolic commitment to serve a detoxification and catabolic function, turning excess D-alanine (beyond what is needed for cell wall synthesis) into a source of nitrogen.

Soil catabolic activities involving D-aspartic acid, D-glutamic acid, D-leucine almost certainly are enabled by induced racemases. In other words, catabolic operons like those in *M. maripaludis* and *S. pombe* but specific for aspartic acid, glutamic acid, and leucine are common in bacteria. The comparable or equal consumption rates of L- and D-enantiomers (Figure 1) suggest that most or all bacteria that consume L-enantiomers also consume D-enantiomers. The contribution of DAAOs cannot be ruled out, but is likely minor, for two reasons. First, yeasts and fungi are present in these soils in minor numbers [34]–[36]. Second, DAAOs are induced by D-enantiomers, not L-enantiomers [21], [24], [37]–[40].

It may seem counterintuitive that catabolic racemases are induced by L-enantiomers. The ecological necessity of this feature becomes evident, however, if we consider the alternative: catabolic operons are induced by D-amino acids only. That is, when encountering racemic amino acids, bacteria “wait” until after L-amino acids are exhausted to synthesize racemases. The organisms would find themselves in a dilemma. To use D-enantiomers, they need racemases. To manufacture racemases, however, they need L-enantiomers, which are no longer available and, without racemases, cannot be supplied by the conversion of D-enantiomers. This is why when only D-enantiomers are added to soils catabolic activities emerge slowly (Figure 2A). The fact that they emerge at all may be because of constitutive alanine racemases. Amino acids racemases are not highly specific. They bind multiple amino acids with varying degrees of efficiency. By expressing catabolic racemases while L-enantiomers are still available, however, bacteria avoid the dilemma and instead transition from using L-enantiomers to using D-enantiomers without interruption.

**Figure 4:**
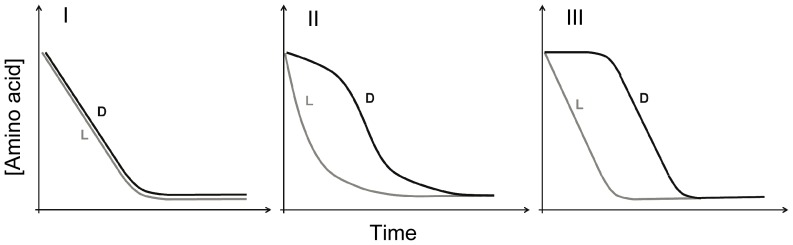
Model-predicted bacterial consumption dynamics of racemic amino acids. Three scenarios occur depending on relative racemase activity: I) the capacity of racemase exceeds that of permease (i.e. *V_R max_* >*V_L max_*); II) the capacity of racemase is equal to or less than that of permease but greater than the excess permeation capacity above the rate of assimilation (i.e. *V_L max_* ≥ *V_R max_* > *V_L max_* –*V_a max_*); and III) the capacity of racemase is less than the excess permeation capacity (i.e. *V_R max_* < *V_L max_* –*V_a max_*).

In racemase-enabled catabolism, D- and L-enantiomers compete with each other kinetically in an asymmetrical manner (Figure S2). Because L-enantiomers are assimilated without conversion, they are always at advantage and can competitively shut down the influx of D-enantiomers, but not vice versa. The application of Michaelis-Menten principles predicts three scenarios (Figure 4; for detailed model description see Text S1 and Figures S3, S4, S5). If the capacity of racemase exceeds that of permease, L- and D-enantiomers are consumed simultaneously and in equal rates (Figure 4, scenario I). This scenario describes the consumption of DL-alanine by wetland and lawn soils. If the capacity of racemase is less than or equal to that of permease, two different scenarios may arise. If the capacity of racemase is greater than the excess capacity of permease above the rate of assimilation, L- and D-enantiomers are imported simultaneously but in unequal rates (Figure 4, scenario II). If the capacity of racemase is less than the excess capacity of permease, the influx of D-enantiomers is prevented. Consumption does not begin until L-enantiomers fall below a critical concentration (Figure 4, scenario III). Most of our soil results and the result with the bacterial isolate *Arthrobacter* sp. conform to scenario II or III.

Available evidence, therefore, warrants reassessment of the generally-held view that the primary biological function of racemases is D-amino acid making. Rather, racemases may have originated for detoxification of D-amino acids from geochemical racemization. Anabolic racemases are likely a secondary biological invention. This evolutionary sequence is more logical, since a detoxification mechanism needs to be in place before a toxin is synthesized for constructive purposes.

Today on Earth, bacteria are a source and also the primary sink for D-amino acids. The latter is a globe-wide ecosystem service. It prevents free-form D-amino acids from accumulating in the environments to toxic concentrations.

## Materials and Methods

Soils were collected from the Mojave Desert (N35°11′37″, W115°49′42″), the Clark County Wetland Park (N36°6′8″, W115°1′18″), and Mount Charleston (N36°16′19″, W115°34′12″) with permission from the Mojave National Preserve (permit# MOJA-00048), the Southern Nevada Water Authority, and the National Forest Service, respectively. No permits were required to collect from the Atacama Desert (S28°28′40″, W70°42′42″) or landscape lawns in Las Vegas (N36°6′47″, W115°8′43″). None of these activities involved endangered or protected species. A soil import permit was issued by the US Department of Agriculture (P526-100813-013).

Bacteria were enumerated by serial dilution and plating on Lauria-Bertani agar. Study medium was prepared in 0.2 M phosphate buffer saline solution. Quantification of amino acid enantiomers followed the high performance liquid chromatography protocol of Zhao and Bada [41]. Briefly, 10 μl of sample supernatant were mixed with 10 μl of 0.2 M sodium borate buffer (pH 9.45) and 5 μl of *o*-phthaldialdehyde and thiol N-acetyl-L-cysteine reagent. After one minute, the derivatization reaction was stopped by addition of 475 μl of 50 mM sodium acetate buffer. Immediately, 20 μl of the derivatized sample were analyzed on an Agilent 1100 HPLC system with a Phenomenex Luna C_18_ column. The mobile phase consisted of methanol and 50 mM sodium acetate, mixed in a gradient program. The flow rate was 1 ml/minute. Column effluent was monitored with a fluorescence detector at excitation wavelength of 340 nm and emission wavelength of 450 nm. Amino acid quantity was calculated from peak area and calibrated against standards. Measurements were reproducible with less than 1% error.

## Supporting Information

Figure S1 **Abiotic adsorption of racemic amino acids (L-enantiomers: open symbol; D-enantiomers: filled symbol) by autoclaved soils.** Unlike biological consumption, this activity is transient and stereo-optically nonselective.
(TIF)

Figure S2 **Schematic diagram of racemase-enabled consumption of racemic amino acids.** D- and L-enantiomers are imported by the same permease. In the cell, D-enantiomers are converted to, and assimilated as, L-forms.
(TIF)

Figure S3 **Model-predicted uptake of racemic amino acids when the capacity of racemases is greater than that of permease.** D- and L-enantiomers are consumed equally.
(TIF)

Figure S4 **Model-predicted uptake dynamics of racemic amino acids when the capacity of racemase is less than or equal to that of permease but greater than the excess capacity of permease above the rate of assimilation.** D- and L-enantiomers are consumed simultaneously but in unequal rates.
(TIF)

Figure S5 **Model-predicted uptake dynamics of racemic amino acids when the excess capacity of permease is greater than the capacity of the racemase.** Consumption of D-enantiomers begins after L-enantiomers are depleted, with rate that may be limited by assimilation (a) or racemization (b).
(TIFF)

Text S1 **Model description.**
(DOC)
