# Supplementary figures and images for: Racemization in Reverse: Evidence that D-Amino Acid Toxicity on Earth Is Controlled by Bacteria with Racemases
Source: PLoS One. 2014 Mar 19;9(3):e92101. doi: 10.1371/journal.pone.0092101 (PMC3960212; doi:10.1371/journal.pone.0092101)

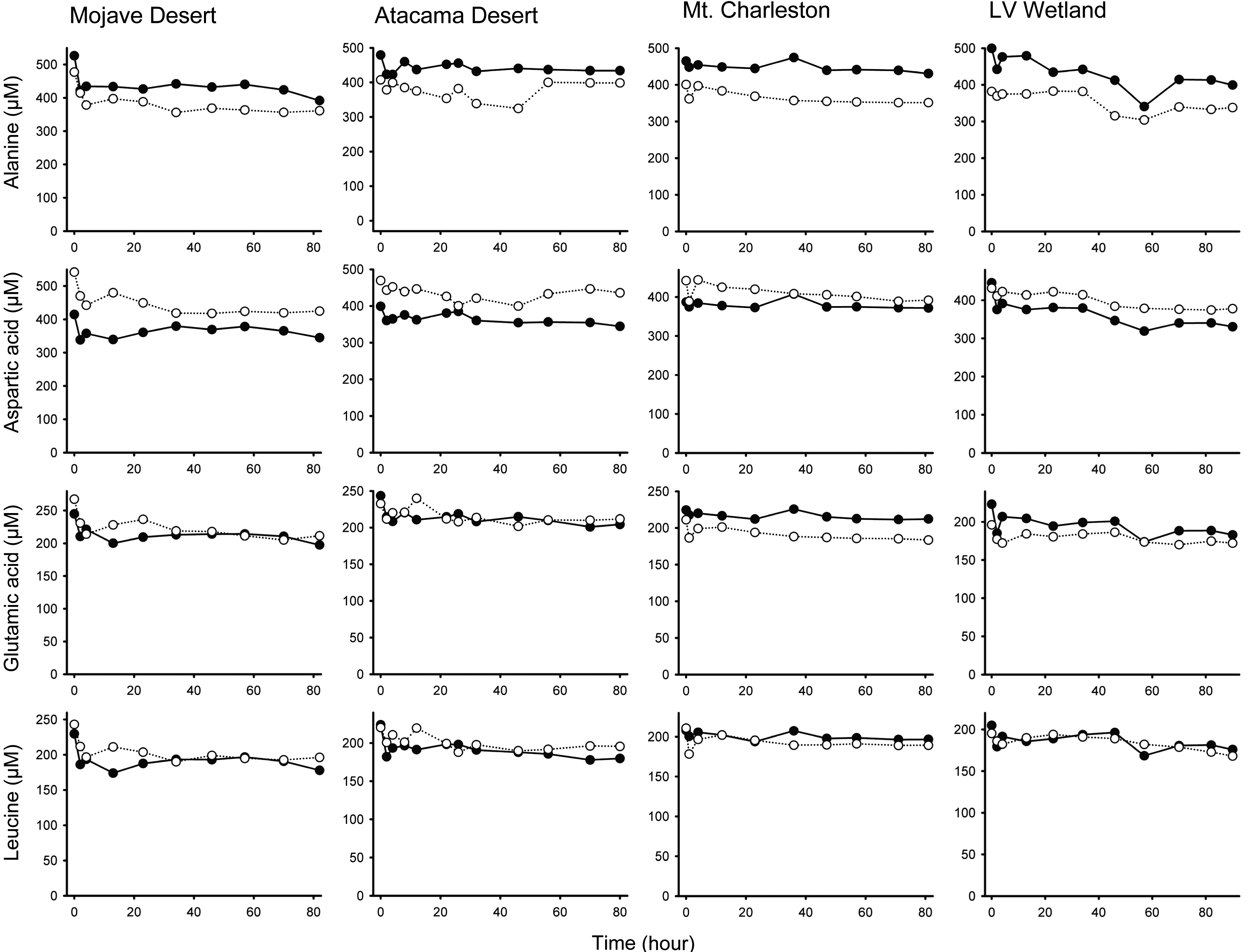

Supplement: Figure S1 — Abiotic adsorption of racemic amino acids (L-enantiomers: open symbol; D-enantiomers: filled symbol) by autoclaved soils. Unlike biological consumption, this activity is transient and stereo-optically nonselective. (TIF) [file pone.0092101.s001.tif]

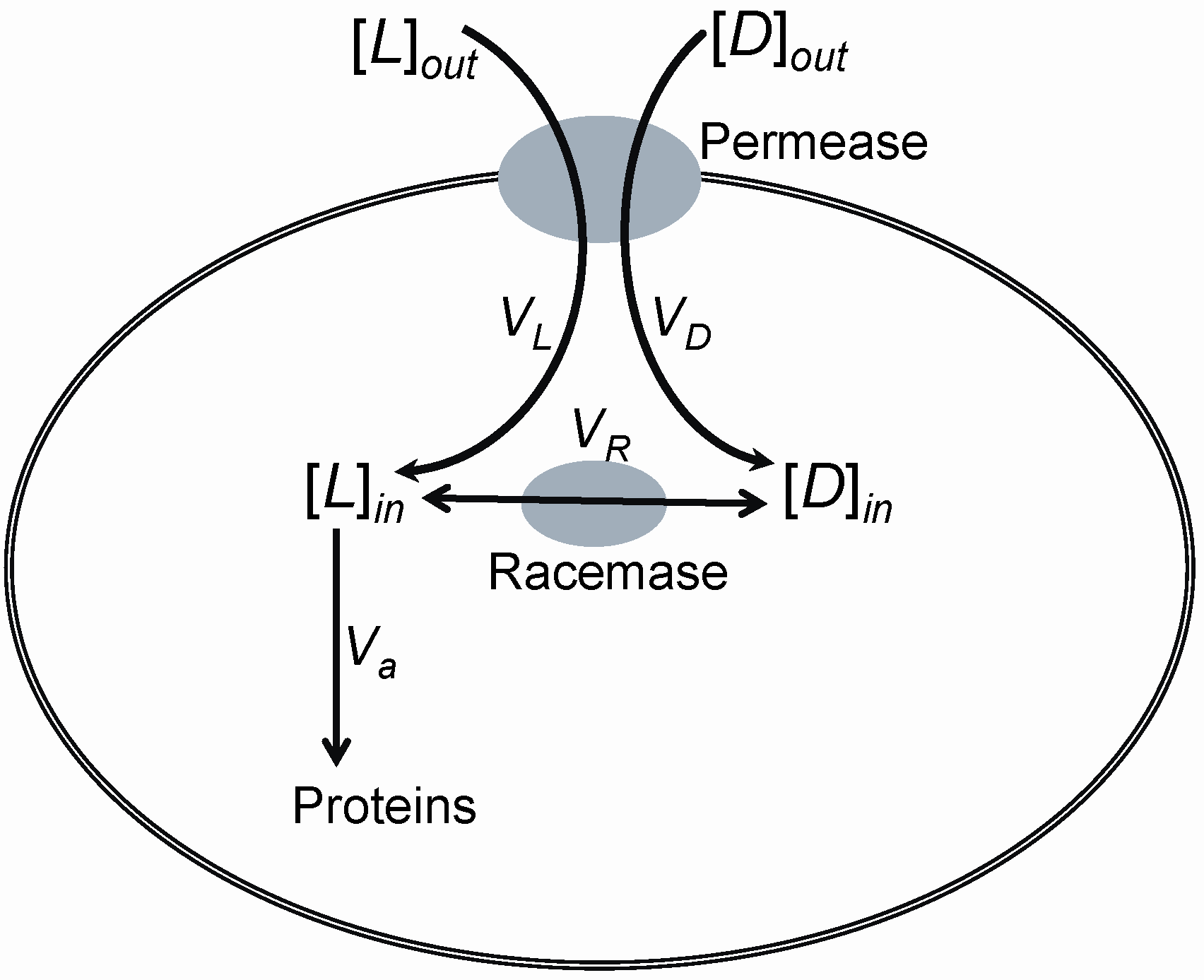

Supplement: Figure S2 — Schematic diagram of racemase-enabled consumption of racemic amino acids. D- and L-enantiomers are imported by the same permease. In the cell, D-enantiomers are converted to, and assimilated as, L-forms. (TIF) [file pone.0092101.s002.tif]

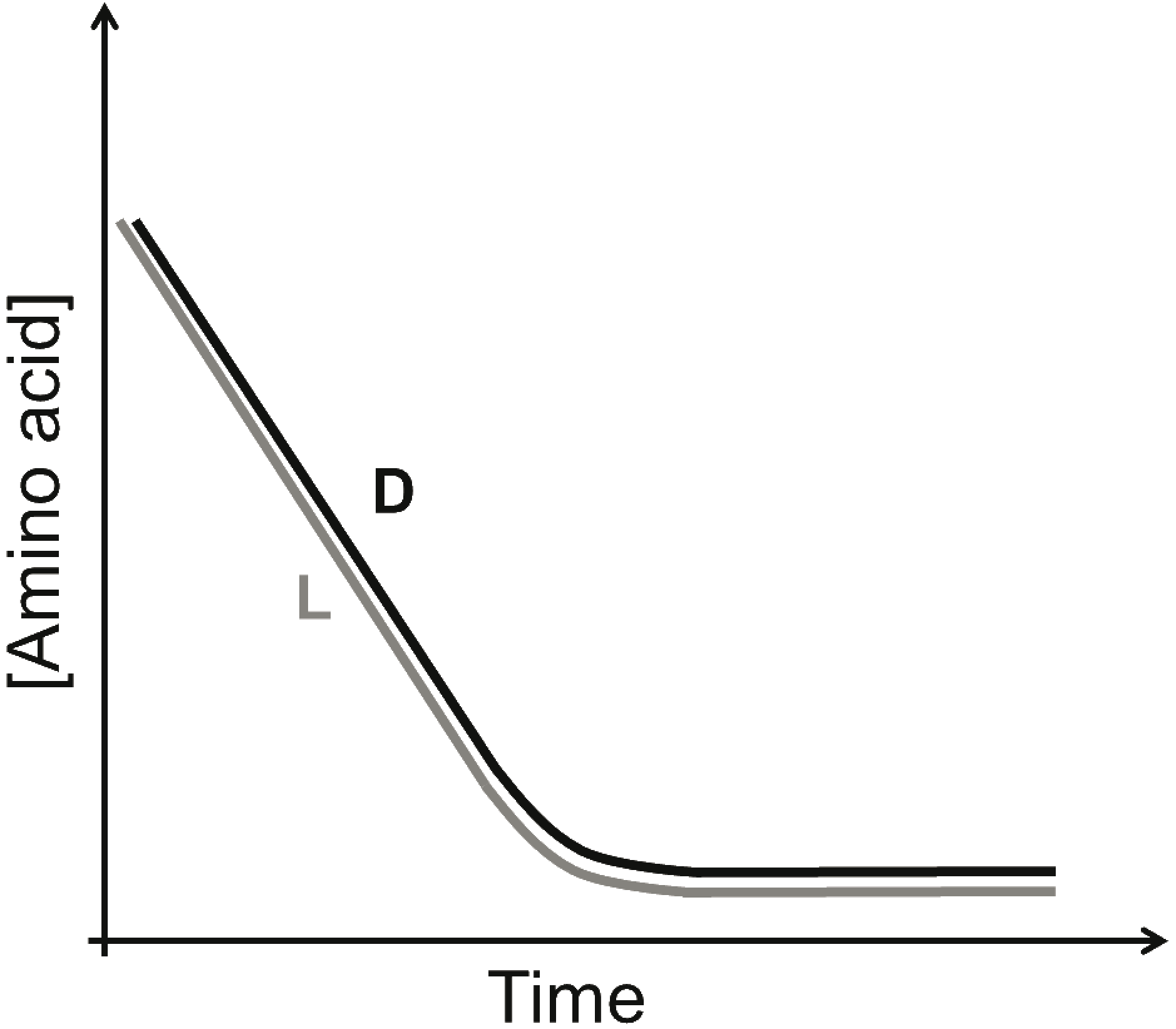

Supplement: Figure S3 — Model-predicted uptake of racemic amino acids when the capacity of racemases is greater than that of permease. D- and L-enantiomers are consumed equally. (TIF) [file pone.0092101.s003.tif]

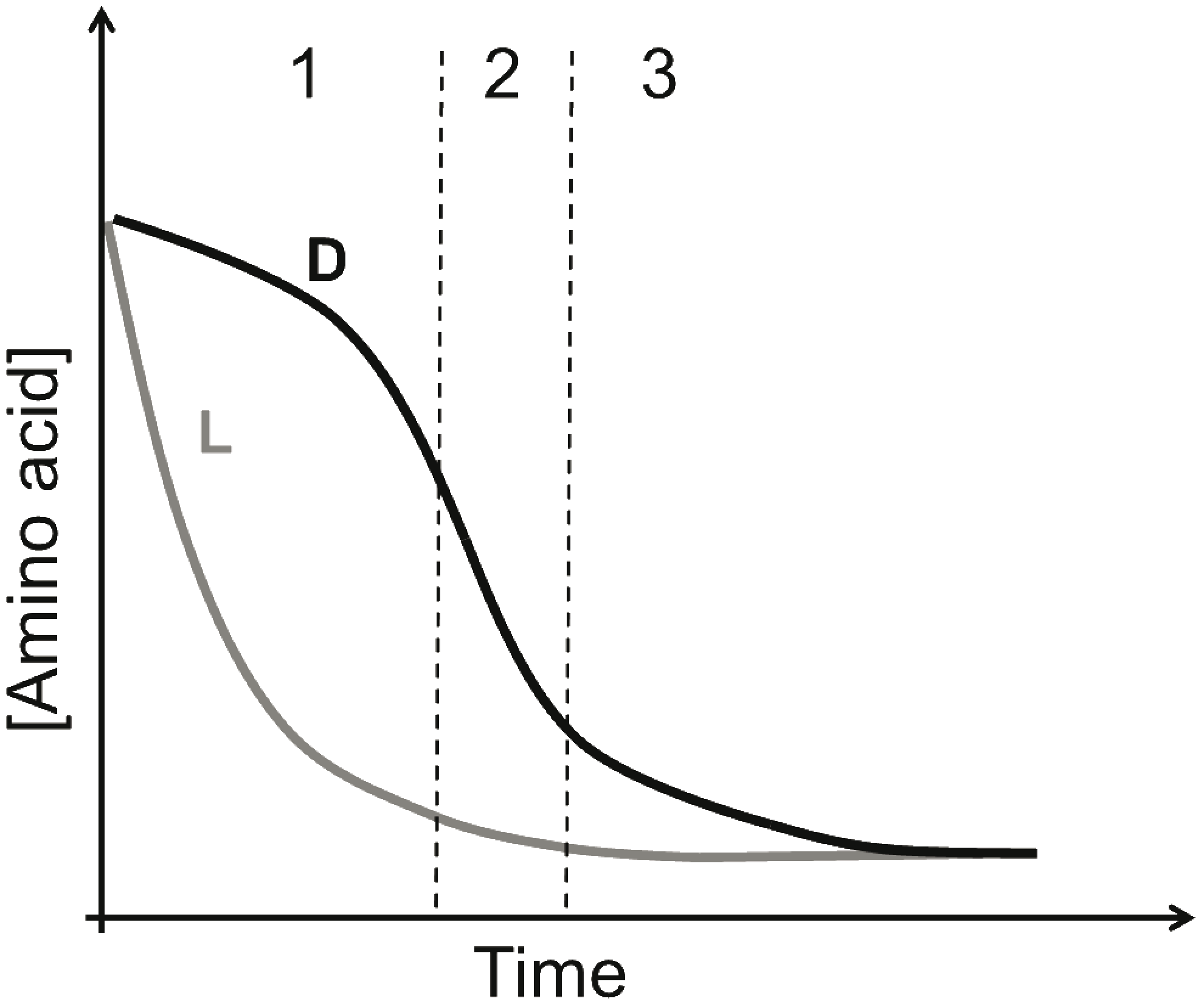

Supplement: Figure S4 — Model-predicted uptake dynamics of racemic amino acids when the capacity of racemase is less than or equal to that of permease but greater than the excess capacity of permease above the rate of assimilation. D- and L-enantiomers are consumed simultaneously but in unequal rates. (TIF) [file pone.0092101.s004.tif]

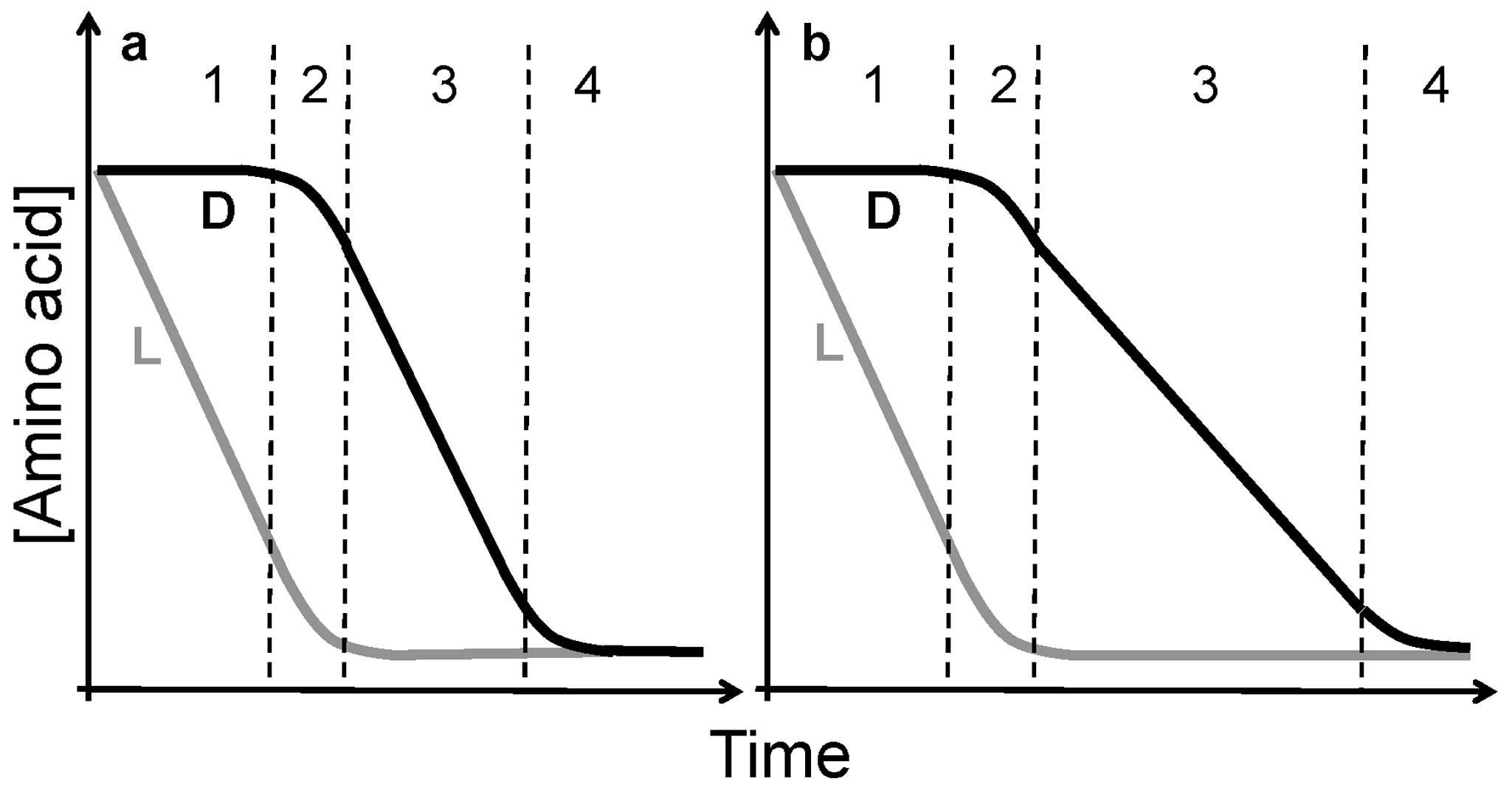

Supplement: Figure S5 — Model-predicted uptake dynamics of racemic amino acids when the excess capacity of permease is greater than the capacity of the racemase. Consumption of D-enantiomers begins after L-enantiomers are depleted, with rate that may be limited by assimilation (a) or racemization (b). (TIFF) [file pone.0092101.s005.tiff]
