# Supplementary material for: Racemization in Reverse: Evidence that D-Amino Acid Toxicity on Earth Is Controlled by Bacteria with Racemases
Source: PLoS One. 2014 Mar 19;9(3):e92101. doi: 10.1371/journal.pone.0092101 (PMC3960212; doi:10.1371/journal.pone.0092101)
Supplement: Text S1 — Model description. (DOC) [file pone.0092101.s006.doc]

M**odel Description**

The diagram in Figure S2 illustrates the early steps in bacterial catabolism of amino acids where it makes a difference whether the amino acid molecule is in D- or L-form. After uptake, an L-molecule is assimilated directly, but a D-molecule has to be converted before it can be assimilated. Because of this difference, the displacement of L-enantiomers by D-enantiomers in the initial uptake of a racemic mixture cannot exceed 50%. The exact percentage is a function of the catalytic capacity of racemase relative to that of permease and of assimilation. If the activity of racemase is not limiting, D- and L-enantiomers will be utilized at the same time and at the same rate. If the activity of racemase is limiting, however, the uptake will change through time from being L-dominated to D-dominated. Below, we describe these different scenarios in detail using these terms:

[*L*]*_out_*, the external L-enantiomer concentration.

[*D*]*_out_*, the external D-enantiomer concentration.

*V_p max_*, the maximum velocity of permease.

*K_m_*, the L-enantiomer concentration corresponding to half of *V_p max_*.

[*L*]*_in_*, the internal L-enantiomer concentration.

[*D*]*_in_*, the internal D-enantiomer concentration.

*V_R max_*, the maximum velocity of racemase.

*V_a max_*, the maximum velocity at which L-enantiomers are assimilated into proteins.

**Scenario I:** D- and L-enantiomers are consumed simultaneously and in equal rates

The capacity of racemase in the cell exceeds that of permease:

*V_R max_* >*V_L max_*

D- and L-enantiomers are consumed identically, contributing 50:50 to the amino acid need of the organism (Figure S3).

$$\begin{matrix} \left[D\right]_{out}^{t}=\left[D\right]_{out}^{0}-\frac{1}{2}v_{a}t \\ \left[L\right]_{out}^{t}=\left[L\right]_{out}^{0}-\frac{1}{2}v_{a}t \end{matrix}$$

$\begin{matrix} \frac{d\left[D\right]_{out}}{dt}=-\frac{1}{2}v_{a} \\ \frac{d\left[L\right]_{out}}{dt}=-\frac{1}{2}v_{a} \end{matrix}$

**Scenario II:** D- and L-enantiomers are consumed simultaneously but in unequal rates

The capacity of racemase is less than or equal to that of permease but greater than the excess capacity of permease above that of assimilation:

*V_L max_* ≥ *V_R max_* > *V_L max_* –*V_a max_*

The influx rate of L-enantiomers is a function of the kinetic potential of permeation, *V_L_*, which, in turn, is a function of [*L*]*_out_*:

The influx rate of D-enantiomers is a function of the kinetic potential of racemization, *V_R_*, which, in turn, is a function of [*D*]*_in_*:

, when [*D*]*_in_* ≥ [*L*]*_in_*

As shown in Figure S4, the consumption of a racemic supply has the following three distinct phases:

*Phase 1*

The combined concentration of D- and L-enantiomers is high enough such that their combined kinetic potential exceeds the kinetic potential of assimilation:

*V_L_* +*V_R_* ≥ *V_a_*

Because L-enantiomers are consumed faster, their kinetic potential, and consequently their influx rate, also decreases faster. The influx rate of D-enantiomers rises accordingly:

$$\begin{matrix} v_{L}=\frac{V_{L}V_{a}}{V_{L}+V_{R}} \\ v_{L}=v_{R}=\frac{V_{R}V_{a}}{V_{L}+V_{R}} \\ v_{L}+v_{D}=v_{a} \end{matrix}$$

During this phase, assimilation occurs at the maximal rate, V_a_.

*Phase 2*

The two enantiomers drop to a level where their combined kinetic potential no longer meets the metabolic demand:

*V_L_* +*V_R_* < *V_a_*

The uptake of D-enantiomers is no longer inhibited. They begin to enter the cell at the rate of , which is controlled by the rate of racemization,. Throughout phase 2,is at its maximum, *V_R max_*.

*Phase 3*

[*L*]*_out_* and [*D*]*_out_* decrease to a point where their influx is limited by the concentration gradient across the cell membrane, not catalysis.

**Scenario III:** D- and L-enantiomers are consumed sequentially

The excess capacity of permease above the cell’s demand is greater than the capacity of the racemase:

*V_L max_* –*V_a max_* >*V_R max_*

The influx of D-enantiomers is prevented. The consumption of a racemic supply proceeds in the following four distinct phases.

*Phase 1*

The excess kinetic potential of permeation above the cell’s demand is greater than the kinetic potential of racemization:

*V_L_* –*V_a_* >*V_R_*

With the influx of D-enantiomers stopped, L-enantiomers enter at the rate of assimilation (Figure S5):

$$v_{L}=v_{a}$$

Therefore,

$$\begin{matrix} \left[D\right]_{out}^{t}=\left[D\right]_{out}^{0} \\ \left[L\right]_{out}^{t}=\left[L\right]_{out}^{0}-v_{a}t \end{matrix}$$

$$\begin{matrix} \frac{d\left[D\right]_{out}}{dt}=0 \\ \frac{d\left[L\right]_{out}}{dt}=-v_{a} \end{matrix}$$

*Phase 2*

The concentration of L-enantiomers is no longer high enough to inhibit the influx of D-enantiomers:

*V_L_* –*V_a_* ≤ *V_R_*

D-enantiomers begin to enter the cell at the rate of enantiomeric conversion, or . The combined influx rate meets the cell’s amino acid demand:

$$\begin{matrix} v_{L}=\frac{V_{L}V_{a}}{V_{L}+V_{R}} \\ v_{L}=v_{R}=\frac{V_{R}V_{a}}{V_{L}+V_{R}} \\ v_{L}+v_{D}=v_{a} \end{matrix}$$

*Phase 3*

As L-enantiomers are exhausted (), their influx rate,$v_{\text{L}}$, approaches zero (,). At this point, the influx of D-enantiomers is controlled by racemization or assimilation, whichever is slower.

If assimilation is slower, *V_R_* ≥ *V_a_*, the rate of influx of D-enantiomers reflects the rate of assimilation (i.e. $v_{D}=v_{a}$)(Figure S5a). Hence,

$$\left[D\right]_{out}^{t}=\left[D\right]_{out}^{0}-v_{a}t$$

$$\frac{d\left[D\right]_{out}}{dt}=-v_{a}$$

If racemization is slower, *V_R_* < *V_a_* , the rate of influx of D-enantiomers reflects the rate of racemization (i.e. )(Figure S5b). Hence,

$$\left[D\right]_{out}^{t}=\left[D\right]_{out}^{0}-v_{R}t$$

$$\frac{d\left[D\right]_{out}}{dt}=-v_{R}$$

*Phase 4*

External D-enantiomers decrease to the point where the influx is controlled by the kinetic potential of permeation:

$$\left[D\right]_{out}^{t}=\left[D\right]_{out}^{0}-V_{D}t$$

$$\frac{d\left[D\right]_{out}}{dt}=-V_{D}$$
